# Improving the Toughness and Thermal Resistance of Polyoxymethylene/Poly(lactic acid) Blends: Evaluation of Structure–Properties Correlation for Reactive Processing

**DOI:** 10.3390/polym12020307

**Published:** 2020-02-03

**Authors:** Jacek Andrzejewski, Katarzyna Skórczewska, Arkadiusz Kloziński

**Affiliations:** 1 Institute of Materials Technology, Polymer Processing Division, Faculty of Mechanical Engineering, Poznan University of Technology, Piotrowo 3, 61-138 Poznan, Poland; 2 Faculty of Chemical Technology and Engineering, UTP University of Science and Technology, Seminaryjna 3, 85-326 Bydgoszcz, Poland; katarzyna.skorczewska@utp.edu.pl; 3 Institute of Chemical Technology and Engineering, Poznan University of Technology, Berdychowo 4, 60-965 Poznan, Poland; arkadiusz.klozinski@put.poznan.pl

**Keywords:** polymer blends, poly(lactic acid), polyoxymethylene, reactive extrusion, material performance

## Abstract

The study focuses on the development of polyoxymethylene (POM)/poly(lactic acid) (PLA) blends with increased impact and thermal resistance. The study was conducted in two phases; in the first part, a series of unmodified blends with PLA content of 25, 50, and 75 wt.% was prepared, while the second part focused on the modification of the PLA/POM (50/50) blends. An ethylene/butyl acrylate/glycidyl methacrylate terpolymer (E/BA/GMA) elastomer (EBA) was used to improve the impact strength of the prepared blends, while reactive blending was used to improve interfacial interactions. We used a multifunctional epoxy chain extender (CE) as the compatibilizer. Static tensile tests and notched Izod measurement were used to evaluate the mechanical performance of the prepared samples. The thermomechanical properties were investigated using dynamic mechanical thermal analysis (DMTA) analysis and heat deflection temperature (HDT)/Vicat softening temperature (VST) methods. The crystallinity was measured using differential scanning calorimetry (DSC) and wide-angle X-ray diffraction (WAXS) measurements, while the rheology was evaluated using a rotational rheometer. The paper also includes a structure analysis performed using the SEM method. The structural tests show partial miscibility of the POM/PLA systems, resulting in the perfect compatibility of both phases. The impact properties of the final blends modified by the EBA/CE system were found to be similar to pure POM resin, while the E modulus was visibly improved. Favorable changes were also noticeable in the case of the thermomechanical properties. The results of most of the conducted measurements and microscopic observations confirm the high efficiency of the reaction for PLA as well as for the modified POM/PLA mixtures.

## 1. Introduction

The use of a polymer mixture based on poly(lactic acid) (PLA) has been one of the dominant trends in the modification of biopolymers and biocomposites over the last few years. The popularity of melt blending technology results from its low cost; typically this method of polymer modification does not require any additional treatments and is conducted at the stage of the preparation of polymer compositions, where the composition of matrix polymers is compounded with other additives, including composite fillers, compatibilizers, and functional additives (nucleating agents and fire retardants, etc.), in one mixing process on a twin screw extruder.

Regarding PLA itself, there are several important reasons for blending this material with other polymers. The first one is the need to facilitate or accelerate the biodegradation process of PLA. This issue is a significant problem in the packaging industry, where biopolymers are expected to have a simple and rapid decomposition process, most preferably under composting conditions. Unfortunately, this polymer normally requires composting under industrial conditions at a temperature of about 80° C. Under normal composting conditions, when the temperature does not exceed the PLA glass transition range of about 60 °C, the depolymerization process is very long and can take up to several years. For these reasons, the process of mixing with other biodegradable polymers seems to be a simple method to accelerate the breakdown and degradation of blends with the addition of PLA. The most popular polymers used for this purpose are polybutylene adipate terephthalate (PBAT) [1,2,3,4], polybutylene succinate (PBS) [5,6], polycaprolactone (PCL) [7,8,9] and thermoplastic starch (TPS) [10,11,12]. Each of these polymers has a low glass transition temperature and a rapid decomposition process under standard composting conditions. The decomposition process of PLA itself, which is often the main ingredient in the blend, also accelerates. This results from the significant fragmentation of the structure of the composted product, as well as the presence of decomposition products of other components of the blend system, which often leads to a change in the pH of the composting environment.

The second category of PLA-based blends includes materials designed to modify the mechanical properties of the base polymer. In this case, the expected features are usually elongation at break or impact strength, which have very low values in pure PLA. In this case, the addition of polymers with elastomeric properties is most commonly used. These can be other biodegradable materials, such as PBAT or PBS; however, the most effective ones are dedicated elastomers designed for the modification of other types of thermoplastic polyesters. The most effective materials are functionalized copolymers that enable the creation of a beneficial core–shell structure, including additives such as ethylene-methyl acrylic-glycydyl methacrylate copolymer (EMA–GMA) [13], ethylene-butyl acrylic-glycydyl copolymer (EBA–GMA) [14], or polyether block amide (PEBA) [15]. In each of these cases, the amount of the required elastomer additive ranges from 20 to 30%. Interestingly, it is then often possible to obtain material parameters that do not differ from those obtained for commercial polymers, including polycarbonate (PC), acrylonitryl-butadiene-styrene (ABS), or their blends (PC/ABS).

The present study investigates the use of PLA as a bio-additive to technical plastics, i.e., in our case, polyoxymethylene (POM). In recent years this problem has gained in popularity, as evidenced by commercial applications of PLA-based blends [16]. The most popular polymer systems of this type include PLA blends with polyamides (PA6, PA610, and PA11) [17,18,19] and polyesters (polyethylene terephthalate (PET), polybutylene terephthalate (PBT), and polytrimethylene terephthalate (PTT)) [20,21,22,23,24]. Research has also been conducted for technical materials such as polycarbonate [25,26,27] and ABS copolymer [28,29,30]. Examples of research into the use of POM as an additive or PLA modifier are not so numerous, but they provide extensive analysis of the issue. One of the most comprehensive publications on this topic is the work of Guo et al. [31,32,33], where the authors present issues related to the miscibility of both polymers. These studies show that for most cases POM and PLA show a high degree of miscibility, which has been confirmed by numerous analyses. Additionally, an analysis of the crystalline phase formation was conducted, which in turn showed that even a small addition of the POM phase may act as an effective nucleating agent for the PLA crystal phase. Similar conclusions have been reached by other researchers dealing with the topic of POM/PLA blends [34,35,36,37]. Interestingly, POM-based blends are not popular in the industry, even though their properties have been the topic of scientific research for many years [38,39,40,41].

Considering literature reports on both POM-based blends and available PLA modification methods, the purpose of this work was to subject POM/PLA blend systems to comprehensive modification. The two main goals for the planned work were to improve the materials’ toughness and increase the thermal resistance. To achieve these goals, the tested materials were subjected to three types of modification. The first one involved the introduction of a rubbery impact modifier, ethylene/butyl acrylate/glycidyl methacrylate terpolymer (E/BA/GMA), a material which has already been successfully applied to PLA-based blends [14,21,42] and POM/PLA blends [31]. The second method was based on the use of reactive blending and phase compatibilization using epoxy functionalized oligomers. This method has also been applied in the modification of PLA-based blends [29,43,44], while the modification of POM-based blends is rarely used [40]. The third type of modification involved annealing thermal treatment. The use of this method is quite common and applies to a wide range of polymer materials, including polyolefins [45,46] and polyesters [7,47,48].

In our preliminary study, unmodified POM/PLA blends were prepared. The POM/PLA content ratios were 100/0 (pure POM), 75/25, 50/50, 25/75, and 0/100 (pure PLA). The purpose of this study was to assess and describe the overall mechanical performance of POM/PLA-type blends and to describe changes in thermomechanical properties for these materials. In the second stage of the work, the balanced POM50/PLA50 blends were subjected to complex modification using E/BA/GMA as an impact modifier. The reactive extrusion process was performed in order to increase the phase interactions, and for this purpose a multifunctional epoxy-based chain extender (CE) was used. The prepared samples were subjected to detailed mechanical tests, thermal analysis (differential scanning calorimetry (DSC) and dynamic mechanical thermal analysis (DMTA)) and rheological measurements. Structural observations were carried out using scanning electron microscopy. The tests were completed using XRD measurements.

## 2. Materials and Methods

### 2.1. Materials

The polyoxymethylene resin used in the course of this study was the acetal copolymer Tarnoform 300 produced by Grupa Azoty (Tarnow, Poland); this type of POM resin is designed for injection molding purposes and has a melt flow index (MFI) of 9 g/10 min (2.16 kg/190 °C). The second polymer used for the preparation of the blends was the poly(lactic acid) Ingeo 3251D produced by Nature Works (Minnetonka, MN, USA); this PLA is a biopolymer made of renewable raw materials for which the manufacturing process is based on the polymerization of lactic acid fermented sugars obtained from plants. Because this grade is intended mainly for the injection molding process, it is characterized by a relatively low molecular weight (88,000 g/mol) and a relatively high MFI of 35 g/10 min (2.16 kg/190 °C). The impact modifier (EBA) used for this study was E/BA/GMA, which was supplied under the trade name Elvaloy PTW (Du Pont, Wilmington, DE, USA). The reactive compounding process was performed in the presence of a Joncryl 4368C reactive compatibilizer (BASF, Ludwigshafen, Germany), which is a multifunctional chain extender based on a styrene–acrylic oligomer functionalized with reactive epoxy groups. According to the manufacturer, the molecular weight of the used chain extender is 6800 g/mol, while the epoxy equivalent reaches 285 g/mol.

### 2.2. Sample Preparation

The preliminary tests conducted without the use of an impact modifier (E/BA/GMA) and chain extender were conducted for the initial assessment of the miscibility of the used polymers and for the purpose of selecting the optimal type of the POM/PLA blend subjected to further modifications. Blends containing 25, 50, and 75% of PLA resin, designated POM/PLA (75/25), POM/PLA (50/50), and PLA/POM (75/25) respectively, were prepared by the extrusion melt blending process. The machine type used was a ZAMAK 16/40 EHD co-rotating twin screw extruder operating at a screw speed of 100 rpm and a maximum temperature of 200 °C. The extruded strand was cooled in a water bath and pelletized to obtain material necessary for the injection molding process, which was used for sample preparation. The injection molding machine used for this study was an ENGEL ES 80/20 HLS; the injection molding temperature was set to 210 °C and the mold temperature was 50°C. During the plasticization stage, the machine screw operated at 150 rpm. The injection speed was 100 mm/s, while the injection and holding pressure reached 1250 and 750 bar, respectively. The cooling stage time was set to 30 s. The processing parameters were fixed for all formulations.

The preparation of modified samples took place at a fixed 50/50% ratio of base polymers and the addition of a 20% impact modifier (E/BA/GMA). For chain extender compatibilized blends the content of CE was 0.5 phr for all samples. Sample annealing was performed using a cabined drier and the thermal treatment was performed at 100 °C for 1 h. All samples were conditioned for 48 h at room temperature, with samples stored in sealed bags before testing.

### 2.3. Characterization

Characterization of the mechanical properties was performed with the use of a universal testing machine model Zwick/Roell Z020. The static tensile test was conducted according to the ISO 527 standard, where type 1A specimens were examined with a cross-head speed of 10 mm/min. Flexural tests (ISO 178 standard) were conducted using the same machine; the cross-head speed in this case was 2 mm/s, and the span distance was 64 mm. Impact resistance was measured with the use of the Izod notched method (ISO 180 standard) using a Ceast 9050 hammer attached to a 5 J pendulum.

Rheological analysis was performed using an Anton Paar MCR 301 rotational rheometer. Parallel plate geometry was applied for the measurements; the gap distance was set to 1 mm for all measurements and the plate diameter was 25 mm. The measurements were performed using the small amplitude oscillatory shear mode. For performing the frequency sweep tests, the pellets were melted at 190 °C, the measurements were conducted at strain γ = 0.5%, and the angular frequency was varied from 100 to 0.1 rad/s. All measurements were conducted in the linear viscoelastic region, which was determined by preliminary amplitude sweep tests.

DMTA analysis was conducted with the use of an Anton Paar MCR 301 rheometer attached to a torsion mode fixture for solid rectangular samples, and the dimension of all samples was 50 × 10 × 4 mm. Viscoelastic properties were measured from 30 to 150 °C and the heating rate was 2 °C/min. The sample strain was 0.01% and the deformation frequency was 1 Hz. The results of the measurements were collected in the form of storage modulus and tan δ plots.

The thermomechanical properties were investigated using two methods. The first method, which measured the heat deflection temperature (HDT), was conducted according to the ISO 75 standard. The heating rate was set to 2 °C/min and the load used was 1.8 MPa. The samples were measured flatwise with a 64 mm span distance. The second method used was the Vicat softening temperature (VST) measurement. For this measurement 4 mm thick short sections of dumbbell specimens were used (ISO 306 standard). The heating rate was again 2 °C/min and the force applied to the flat-ended needle was 10 N. Testlab RV300C was used for the thermomechanical tests. The tests were performed in a heated oil bath.

The structure of the prepared blends was investigated using the SEM. In order to prepare the surface for observation a dumbbell specimen was cryo-fractured under liquid nitrogen. Then, the fractured surface was sputter-coated with a thin layer of gold. The analysis was conducted using the scanning electron microscope Carl Zeiss EVO 40.

The DSC measurements were carried out using a Netzsch DSC 204 F1 Phoenix apparatus. The specimens for the test were cut from the middle section of the dumbbell sample and the average weight of the single measurement sample was 5 mg. The temperature program consisted of three standard stages (heating/cooling/heating) and the tests were conducted from 30 to 230 °C with a heating/cooling rate of 10 °C/min. All samples were placed in aluminum pans and the heating chamber was purged with nitrogen. The crystallinity of the PLA and POM was determined according to the following equation, Equation (1), i.e.,

(1) $$\%Crystallinity=X_{c}=100\times \frac{\Delta H_{m}-\Delta H_{cc}}{\Delta H_{m}^{0}\left(1-\varphi \right)}$$

where $\Delta H_{m}$ is the measured melting enthalpy, $\Delta H_{CC}$ is the measured enthalpy of cold crystallization, and $\Delta H_{m}^{0}$ is the theoretical melting enthalpy of a 100% crystalline structure. The amount of the secondary phase is expressed by φ. The value used for the theoretical melting enthalpy for poly(lactic acid) was 93.7 J/g [49,50], while for POM it was 326 J/g [51,52].

The crystalline structure of the obtained materials was also investigated by wide-angle X-ray diffraction (WAXS). These tests were conducted with the use of a URD 6 diffractometer (Seifert FPM, Germany) using the step method with a measuring system in Bragg–Brentano geometry in the 2θ angle range from 10 to 80 with a step of 0.1. An X-ray tube with a copper anode was used (CuKα, wavelength of λ = 1, 5406 Å) as the X-ray source. XRD measurements were conducted on the injection-molded samples (on the parallel central part of the samples) which had been previously conditioned in the manner described above.

## 3. Results

### 3.1. Mechanical Properties (Static Tensile Test and Izod Notched Measurements)

**Figure 1:**
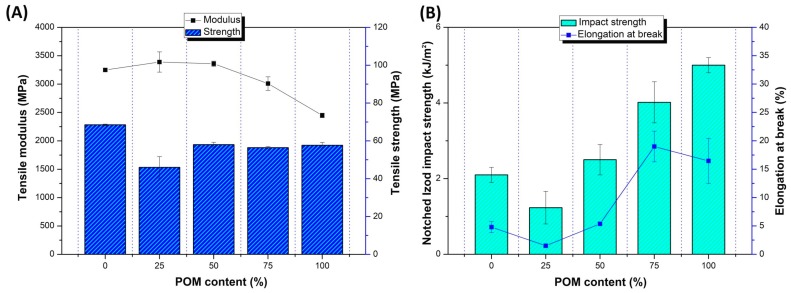
The mechanical properties of unmodified blends and reference polyoxymethylene (POM) and poly(lactic acid) (PLA) samples. Tensile modulus and strength (**A**); elongation at break and Izod notched impact strength (**B**).

The results of the measurements from the static tensile and Izod impact tests are shown in Figure 1. Tensile modulus and strength (Figure 1A) are compared to elongation at break and impact strength values (Figure 1B); the comparison refers to preliminary unmodified blends where the chain extender and impact modifier were not used. As expected, most of the mechanical parameters followed the rule of mixture tendencies. As the differences between the tensile strength and modulus of pure POM and PLA are not large, the results for POM/PLA do not show any significant fluctuations for these values. Slightly more significant differences can be seen to occur for the elongation at break and impact strength, where the growing content of POM resin leads to a visible increase in both factors for POM-rich blends.

**Figure 2:**
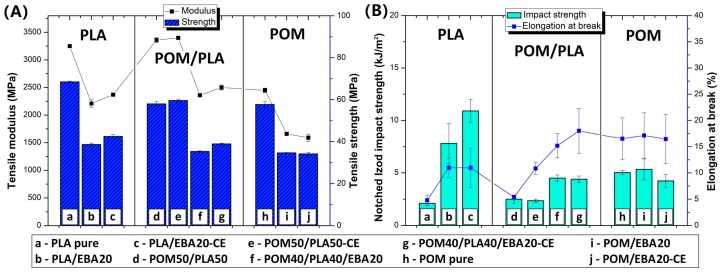
A comparison of the mechanical properties of the prepared blends from tensile and impact resistance measurements for the “as-molded” samples. (**A**) Tensile modulus and strength. (**B**) Elongation at break and impact strength. Results are grouped into PLA-, POM-, and POM/PLA-based samples modified using the ethylene/butyl acrylate/glycidyl methacrylate terpolymer (E/BA/GMA) elastomer (EBA) impact modifier and epoxy-functional styrene acrylic copolymer (ESA) chain extender (CE).

**Figure 3:**
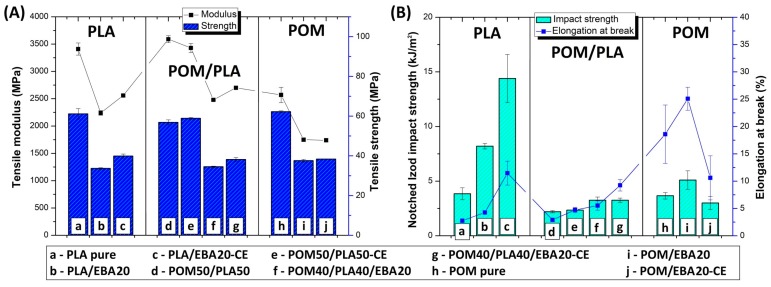
A comparison of the mechanical properties of the prepared blends with regard to the tensile and impact resistance measurements for the “annealed” samples. (**A**) Tensile modulus and strength. (**B**) Elongation at break and impact strength. Results are grouped into PLA-, POM-, and POM/PLA-based samples modified using the E/BA/GMA impact modifier and ESA chain extender.

Preliminary results confirm the predictable nature of changes in the mechanical properties of the POM/PLA blends. The balanced POM/PLA (50/50) blend was selected for further modification using an impact modifier and chain extender. The samples from pure POM and PLA were subjected to a similar modification procedure for comparison. In addition to the blending processes, all the EBA-modified samples were subjected to an annealing procedure (100 °C, 1 h); the choice of temperature and annealing time was selected based on the literature and our own experience [36,53,54]. The results of the mechanical test are presented in Figure 2 and Figure 3 for the as-molded and annealed samples, respectively. The results of tensile strength and E modulus for the as-molded POM/PLA blends reveal a behavior typical for impact modified materials. After the introduction of the elastomeric EBA the stiffness and strength of the investigated POM/PLA blend can be seen to radically drop. For reference, in the POM and PLA samples the same amount of 20% EBA was also found to cause a decrease in tensile strength and modulus by about 30 and 40%, respectively. Similar results were observed for both the pure POM and PLA samples after the addition of rubbery impact modifiers.

A similar trend was observed for the annealed samples; however, due to the increased crystallinity level the initial values of the tensile modulus for the POM/PLA blend, the pure POM, and the pure PLA were visibly higher. The more significant changes relate to the results of elongation at break and impact strength, where both characteristics for the unmodified POM/PLA blend were found to be at a similar level to pure PLA, clearly indicating the need for further modification. The introduction of the epoxy CE, EBA impact modifier and combined CE/EBA system caused a large improvement in the elongation at break. The value of this moved from an initial value of 5.4%, to 10.8% (blend+CE), to 15.2% (blend+EBA), and to 18.0% (blend+CE/EBA), with the final values being very close to the elongation values reported for pure POM (16.5%). However, for the POM samples the introduction of CE and EBA did not lead to any visible changes in elongation values. Unlike with the pure POM samples, the modification of PLA resulted in a large increase in the elongation value, from an initial 4.8% to 11% for both the EBA and EBA/CE systems. Considering the impact resistance of the POM/PLA blends, visible changes are reported only for the EBA-modified samples: 4.5 J/m for the EBA-modified blend (POM40/PLA40/EBA20) and 4.4 J/m for the EBA/CE-modified sample (POM40/PLA40/EBA20-CE). The introduction of CE into the blend did not improve the impact strength, while the value of 2.3 J/m for the POM50/PLA50-CE sample can be seen to be very close to that of the reference unmodified POM/PLA blend (2.5 J/m). In terms of the elongation at break, the impact strength of the POM-based samples remained at an identical level for the reference and modified samples. The introduction of the modifiers was more favorable for the pure PLA samples, where the combined EBA/CE system led to the largest improvement if considering all the molded samples. The initial PLA impact strength of 2.1 J/m was increased by five for the PLA/EBA20-CE samples (11 J/m). Interestingly, the annealing treatment led to a further improvement of up to 14.5 J/m; however, the positive changes after the thermal treatment applied only to the pure PLA samples, while the properties of the POM/PLA blends deteriorated significantly, both for impact strength and elongation at break.

The results of the impact tests emphasize the different behaviors of the individual base polymers. Comparative tests for pure polymers show that PLA is much more susceptible to modification, while the POM modification appears to have very limited effectiveness. The most important results were obtained for POM/PLA. Thanks to the use of the modified systems it was possible to obtain very good results for tensile strength, elongation at break, and impact strength, where most of the characteristics were found to be very close to that of pure POM resin. The addition of PLA caused a significant increase in sample stiffness, which can be considered an advantage for this type of blend. The application of the annealing procedure caused the deterioration of most mechanical properties. However, the thermal treatment was applied in order to increase the heat resistance and the reduction in other characteristics was expected.

### 3.2. Rheological Measurements

**Figure 4:**
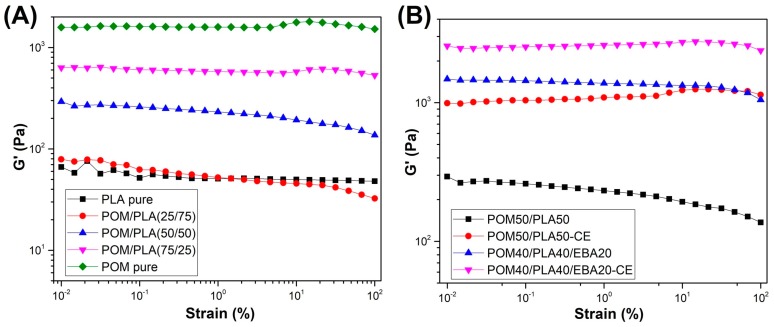
Strain sweep measurement plots for unmodified POM/PLA blends (**A**) and modified POM50/PLA50 blends (**B**).

Rheological measurements were conducted using the rotational rheometer working in the dynamic mode (small amplitude oscillation shear). In order to determine the linear viscoelactic region (LVR) of the prepared blends a strain sweep measurement was performed. As can be observed in Figure 4, for most of the samples the LVR reached at least 10%, which is why the frequency sweep measurements were performed at 1% strain.

**Figure 5:**
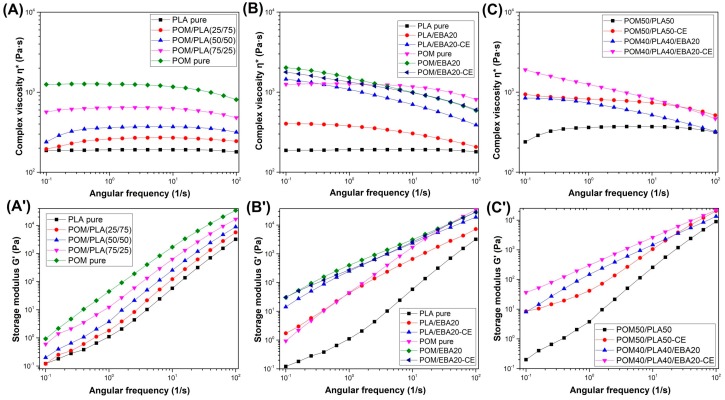
Complex viscosity and storage modulus of unmodified POM/PLA blends (**A**,**A′**), EBA/CE-modified POM and PLA (**B**,**B′**), and modified POM50/PLA50 (**C**,**C′**) samples.

Figure 5 shows complex viscosity η* and storage modulus G* results for the different types of prepared samples based on the unmodified POM/PLA blends and EBA/CE-modified materials. For comparison, all curves have been plotted using the same scale, meaning that the influence of different types of material modifications can be directly compared.

The preliminary tests were conducted on unmodified POM/PLA blends (Figure 5A). For all blends the complex viscosity values ranged between the plots of pure PLA and POM resin. The complex viscosity for PLA can be seen to be constant across almost the whole frequency range of the test, which clearly indicates a matrix thermal decomposition process. The addition of the POM phase increases the viscosity values; however, it does not improve the thermal stability of the PLA matrix, which is manifested by the curve inflection at low deformation frequencies. The main reason for this behavior is the relatively long measurement time of about 12 min, which is unobstructed even in a protective nitrogen atmosphere. The storage modulus variations (see Figure 5A′) show a continuous enhancement, indicating the predominantly elastic response of the material. The increasing interaction between the entangled polymer chains cause deformation restriction, leading to the higher elastic response.

The rheological analysis of the EBA-modified samples for the POM samples indicates very little impact of the presence of the elastomeric phase; neither does the additional use of the chain extender have any visible effect on the viscosity curve. The only visible change involves a slight deviation in the η* curve shape caused by the relaxation of the dispersed phase; however, this phenomenon is typical for all polymer blends and does not indicate chemical interactions between POM and its modifiers [38,55]. The modification of the PLA-based materials offers many more observations. The best results were observed for the modified samples subjected to the reactive extrusion process. Throughout the entire measurement range, the PLA viscosity is maintained at the highest level, and the decrease in the final stage of the test is relatively small and indicates a significant increase in the molecular weight of PLA. The viscosity changes in the lower frequency range may also suggest the appearance of chemical bonds at the PLA–EBA interface [56,57,58].

The viscosity changes for the obtained POM/PLA blends mostly reflect the differences occurring for the PLA-based samples. The unmodified POM/PLA (50/50) blend is characterized by decreasing viscosity. Hence, it seems that the final increased viscosity for the modified POM/PLA blend results from the overlap of two relationships. The first of these is an increase in the PLA viscosity caused by the chain extension and the second is related to the presence of the EBA modifier. Similarly to PLA, the addition of the EBA impact modifier leads to some improvement in the complex viscosity, however, the addition of the CE and EBA/CE system seems to be the most effective with regard to viscosity improvement. The addition of the CE itself indicates a significant increase in the PLA molecular weight, while the subsequent addition of the impact modifier causes an additional binding of the matrix chains at the boundaries of the EBA phase. Thus, the relaxation of the elastomeric EBA phase is hindered, increasing the viscosity.

**Figure 6:**
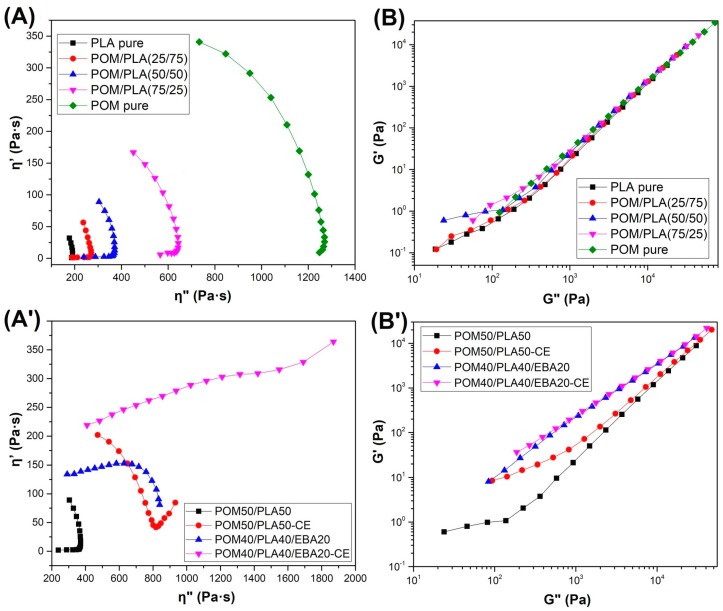
Cole–Cole plots (**A**,**A′**) and Han plots (**B**,**B′**) for unmodified POM/PLA blends and EBA/CE-modified POM50/PLA50 blends.

The phase morphology of the prepared blends was characterized using the Cole–Cole and Han plots (Figure 6). The first type of chart presenting the imaginary viscosity (η″ = G″/ω) versus real viscosity (η′ = G′/ω) values displays semicircular-shaped curves of unmodified POM/PLA blends. This type of behavior confirms the good compatibility between blend phases, which also confirms the miscibility of these polymers in the molten state [59,60]. However, for EBA/CE-modified blends the Cole–Cole plots show a large deviation from the semicircular smooth shape. The presence of the rubbery elastomer and reactive extrusion change the plot shape to a circular arc with a tail, or even a linear shape for the POM40/PLA40/EBA20-CE sample. This type of curve shape indicates the appearance of uncompatibilized phases such as droplet inclusions [61]. The phase compatibility was also able to be evaluated using Han plot analysis (Figure 6B,B′). According to the authors of this conception, Han et al. [62], deviation in the plot slope (G′ versus G″) indicates blend incompatibility. The analysis for the unmodified POM/PLA blends indicates high compatibility/miscibility of these materials. The same analysis for EBA/CE-modified materials reveals significant deviations in plot slope which confirm the presence of uncompatibilized phases.

### 3.3. Thermomechanical Properties—DMTA Analysis and Heat Resistance Tests (HDT and VST)

**Figure 7:**
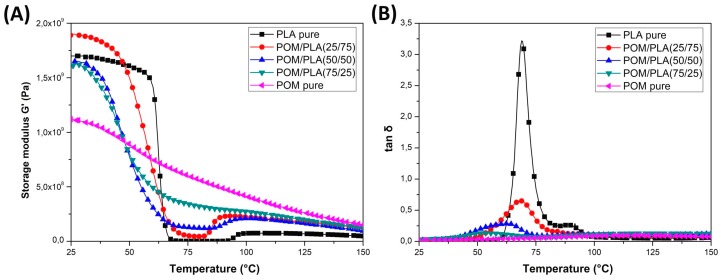
The results of the DMTA measurements for the POM/PLA blends. (**A**) Storage modulus and (**B**) tan thermograms.

DMTA analysis was conducted mainly to assess changes in the thermal resistance of the obtained materials and possible changes in the phase transitions. The thermograms presented in Figure 7 show a comparison of the POM and PLA samples and the unmodified POM/PLA blends. The graphs show the storage modulus and tan δ plots for the as-molded samples. A direct comparison of the G′ plots for POM and PLA reveal a significant difference in the curve characteristics which is mainly related to the sharp decrease in the module’s value for PLA within the glass transition region at around 60 °C. For the POM-based samples, the stiffness changes are less pronounced and mostly result from a high level of crystallinity; no significant changes are recorded in the tested range because the glass transition temperature for POM is observed at around −70 °C.

The DMTA thermograms characterizing the properties of the unmodified POM/PLA blends present a fairly typical trend of changes, especially within the PLA glass transition temperature. Along with the increase in the POM content in the blend, the nature of the glass transition area changes; a characteristic sharp decrease in the stiffness for pure PLA shifts to a more gradual transformation already at 50% POM content. Interestingly, the glass transition temperature range, read from the tan δ charts, also changes. The original value reported for pure PLA at 70 °C gradually shifts to lower values. For the POM/PLA (75/25) blends, the glass transition temperature is 53 °C. Such a change confirms the partial miscibility of the used polymers, which has already been reported in the literature [33,34]. Unusual phenomena include a slight increase in stiffness, expressed by a higher value of the storage modulus, for the PLA/POM (75/25) blend. Given the much lower stiffness for pure POM, all diaphragms should present intermediate values, while the addition of 25% POM improves the storage modulus. The stiffness for the POM/PLA (50/50) and POM/PLA (75/25) samples is only slightly lower than the values presented for pure PLA. The occurrence of this type of change confirms the ability to nucleate the PLA crystal structure by the addition of POM, as reported by Guo et al. [31].

**Figure 8:**
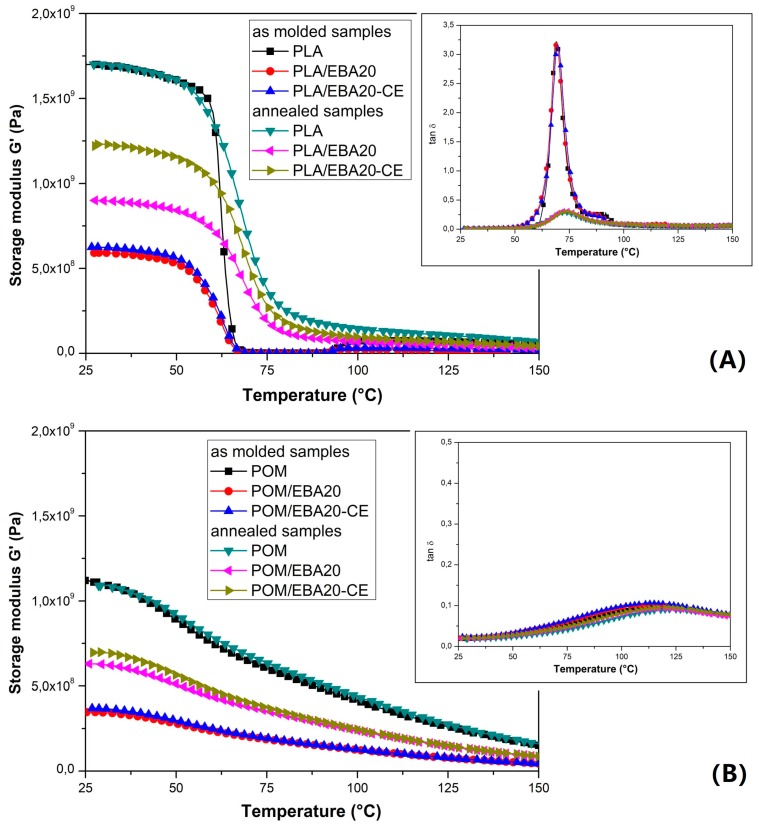
Storage modulus and tan δ thermograms for PLA- (**A**) and POM- (**B**) based samples modified with the E/BA/GMA elastomer and ESA chain extender. The plots include the thermograms of the “as-molded” and “annealed” samples.

**Figure 9:**
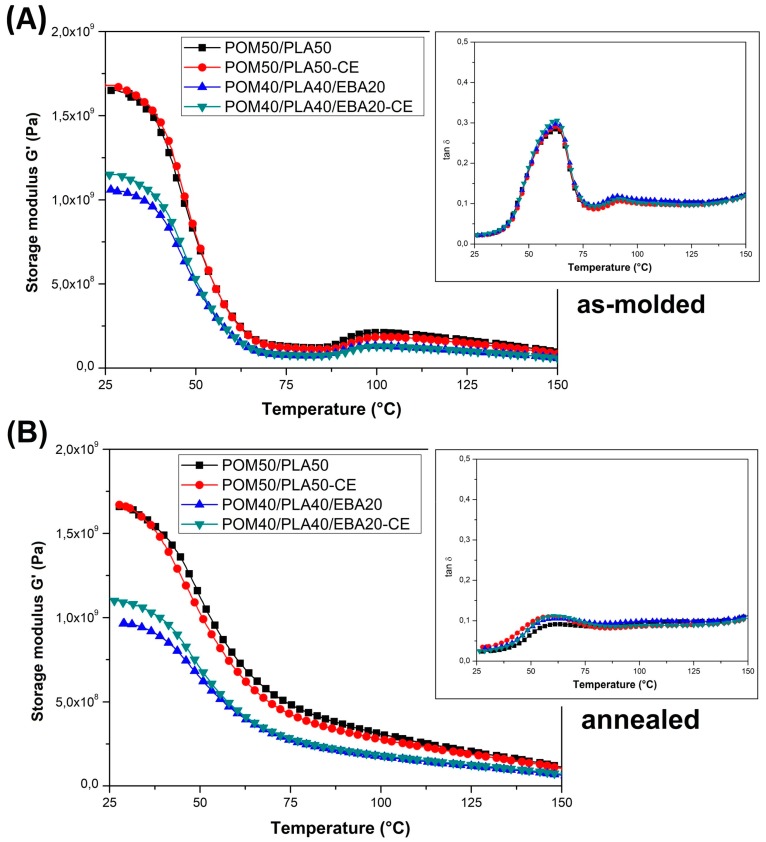
Storage modulus thermograms for the POM/PLA blends. Injection molded samples (**A**) and annealed materials (**B**).

The influence of the modification on the viscoelastic properties of the pure PLA and POM samples is reported in Figure 8, while that for the POM/PLA blends is shown in Figure 9. The storage modulus plots reveal clearly that the stiffness of both polymers is strongly affected by the addition of the EBA modifier, as well as for the annealed specimens. The G′ curves clearly indicate the lack of impact of the annealing process on the POM samples, which is caused by the initial high degree of crystallinity after the injection process.

In turn, for PLA an increase in thermal resistance is clearly noticeable. Before the thermal treatment the values of the G′ modulus for pure PLA drop below 1 MPa, which suggests that the glass transition range determines the final range of application. In contrast, for the annealed samples the stiffness decrease is not rapid and even above 100 °C the G′ values are maintained at a relatively high level. It is clearly visible from the tan δ thermograms that the annealing process significantly reduces the area under the curve of the tan δ plot, indicating a reduction in the amorphous phase in the PLA structure.

The storage modulus thermograms for the POM/PLA blends are presented in Figure 7; the graphs also include the tan δ plots. It is clear that for the prepared blends the results of the DMTA measurements confirm the large impact of miscibility for the POM-PLA system, both on the mechanical properties and the phase transition occurrence. The addition of POM primarily reduces the rapid decrease of the G′ modulus close to the T_g_ temperature; therefore, even for the samples not subjected to the annealing process, the stiffness does not drop to such a critical level as for the PLA-based samples. The annealing treatment also increases thermal resistance, which suggests that this improvement for the untreated blends is mostly caused by the presence of the highly crystalline POM structure rather than the possible nucleation of the PLA crystalline phase.

For the POM/PLA blends, a significant reduction in the area under the tan δ plot indicates a lower content of the PLA amorphous phase, which could have been caused by the induced nucleation effect. The introduction of an impact modifier did not cause any additional changes in the appearance of the curve in the glassy state. The most important observation concerns the glass transition temperature, where the initial peak for the pure PLA at around 70 °C is shifted to 63 °C, suggesting a partial miscibility of the main components of the POM/PLA blend system.

The thermal resistance of the prepared samples was determined with the use of two different methods: HDT and VST (Table 1). For both tests the resulting temperature shows a stiffness decrease around the glass transition region or melting point of the investigated material. The results of the HDT test reveal a large difference of around 30 °C between the pure POM and the PLA resin. Interestingly, the introduction of the annealing procedure did not change the difference between these two polymers; however, for both of them the HDT was improved by ~15 °C. It can be seen that for most of the unmodified POM/PLA blends the HDT is lower than that for the pure POM and PLA. This type of behavior suggests that even a large amount of the highly crystalline POM phase does not increase the resistance to deflection of the material. The annealing procedure causes a clear increase in the HDT values, which in this case is mainly caused by the increase in the PLA crystallinity. Considering the result of the second VST test, the application of the blending procedure can be observed to have resulted in a very clear increase in the softening point for all the materials with the POM addition. For the pure PLA resins the annealing procedure was also very efficient, and the VST was increased from 66 to 152 °C. For all the impact-modified POM/PLA blends the VST exceeded 140 °C. Considering the large differences in the results for the used research methods, it should be emphasized that HDT is a basic test for technical/engineering plastics. From this perspective, only the annealing procedure brings forth a clear increase in the thermal resistance for the POM/PLA blends.

### 3.4. Structure Evaluation

Structure observations were conducted using the scanning electron microscopy method. The initial tests performed on the unmodified POM/PLA samples revealed that the fracture surface for all blends was smooth, with no visible inclusions of the minor phase. To some extent this observation confirms the partial miscibility or good compatibility of the blended polymers. Since the other experiments did not show full miscibility, the phase separation could appear at a very small scale, which was unavailable for the SEM technique.

**Figure 10:**
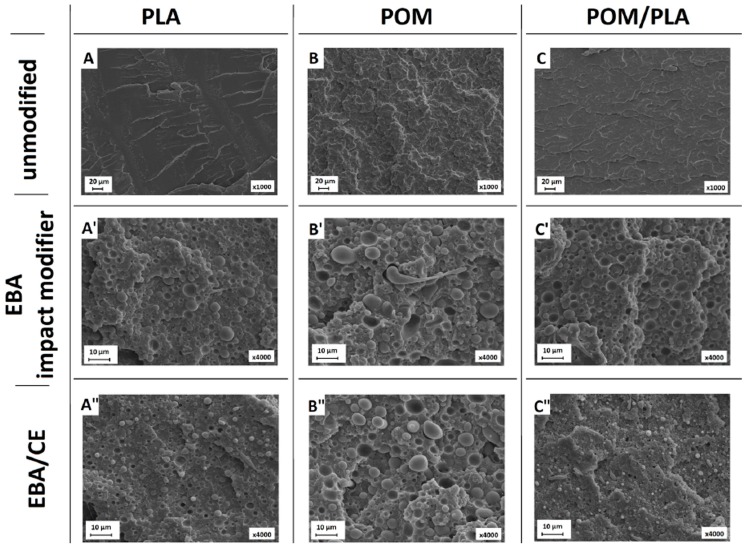
Micrographs presenting the cryo-fracture surfaces of the PLA- (**A**,**A′**,**A″**), POM- (**B**,**B′**,**B″**), and POM/PLA- (**C**,**C′**,**C″**) based samples. Subsequent rows indicate the presence of the unmodified samples (top), EBA-modified samples (middle), and EBA/CE-system-modified blends (bottom).

A comparison of the modified samples can be seen in Figure 10, where the EBA- and EBA/CE-modified samples are compared to the unmodified POM, PLA, and POM/PLA (50/50) samples. The presence of the sea–island structure is confirmed for all the samples; however, the different size in the EBA modifier inclusions suggests a change in the EBA–matrix interactions. The largest inclusions can be observed for the POM-based samples. Interestingly, the addition of CE did not influence the size of the EBA domain, which confirms the lack of chemical interactions during the reactive extrusion of POM/EBA blends. The structure observations are confirmed by the other performed experiments, in which the presence of weak interphase bonds for the modified POM resin is revealed.

In comparison to the POM/EBA samples, the size of the EBA inclusions for the PLA samples is visibly smaller. Moreover, the addition of CE reduced this size significantly. This behavior confirms the higher tendency of PLA to react with the GMA groups present in both the EBA impact modifier structure and the used chain extender. This mechanism was also observed for the POM/PLA blends, where the size of the EBA inclusions for the POM40/PLA40/EBA20 was very close to the PLA/EBA20 sample. An analogous relationship can be seen to occur after the introduction of CE, where similarly to the PLA/EBA20-CE sample the sea–island-like structure was highly dispersed for the POM40/PLA40/EBA20-CE sample.

**Figure 11:**
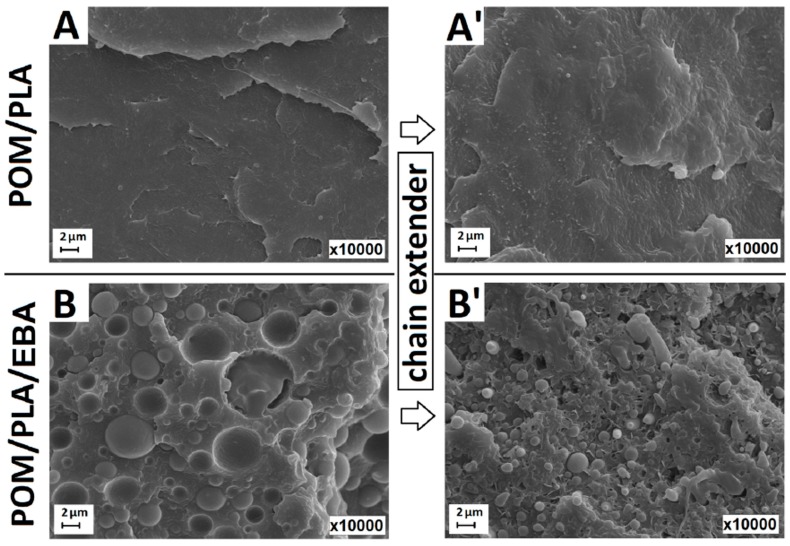
SEM micrographs presenting the cryo-fractured structure of the pure POM/PLA blend (**A**,**A′**) and EBA-modified blends (**B**,**B′**). A and B present samples without CE addition while A′ and B′ show structures following the introduction of CE.

The SEM micrographs in Figure 11 present the higher magnification of the fractured surface (×10,000). The pictures show the structures of the POM/PLA and POM/PLA/EBA blends, before and after the introduction of CE. It can be seen that for the unmodified blend the smooth surface partly confirms the self-compatibilization of both main components; a similar POM/PLA structure appearance has been obtained by other researchers [34]. For the EBA-modified samples the size of the elastomer inclusion was reduced from 3.2 (± 1.3) µm for the POM40/PLA40/EBA20 sample to 0.6 (± 0.3) µm for the CE-modified sample.

### 3.5. Crystallinity Analysis—DSC and WAXS Measurements

DSC analysis was carried out while maintaining the standard heating–cooling–heating procedure, which allowed the determination of the temperature of the phase transitions and the level of crystallinity for individual samples.

**Figure 12:**
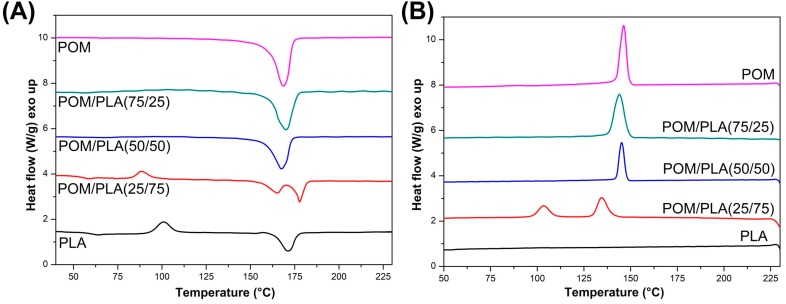
The results of differential scanning calorimetry (DSC) measurements for POM/PLA blends: (**A**) first heating thermograms and (**B**) cooling thermograms.

The results of the preliminary study conducted on the unmodified POM/PLA blends are presented in Figure 12, which shows the first heating and cooling thermograms. A comparison obtained from the heating stage reveals that the melting peak of the POM and PLA resin overlap. The peak maximum temperatures are 171.4 °C and 168.6 for PLA and POM, respectively. For the POM/PLA (75/25) and (50/50) blends the overall appearance of the curve does not differ from the pure POM thermogram; however, for the POM/PLA (50/50) sample a small drop in the melting peak temperature can be noted. The analysis confirms the domination of the thermal effects of the highly crystalline polyoxymethylene phase. The same conclusion refers to the cooling stage plots, where both the peak maximum and peak onset characteristics are very close to the reference POM. A different behavior can be observed for the PLA-rich POM/PLA (25/75) sample. Due to the large amount of the PLA phase an exothermic peak close to 90 °C is detected. The appearance of this peak confirms the cold crystallization of the amorphous PLA structure; however, for the pure PLA sample this phenomenon occurs at 100 °C, which suggests some nucleating effect resulting from the addition of the POM phase. A similar behavior was noted by Guo et al. [32]; in their study the nucleation effect was reported even at 5% POM content. Significant changes in the thermal behavior of the PLA-rich materials are reflected during the melting of the crystalline structure, which is confirmed by the presence of the double melting peak. According to other researchers [31,32,33] the presence of two peaks could be related to the occurrence of two phenomena: partial miscibility and POM nucleation ability. The occurrence of the first melting peak at 165 °C could be related to the miscibility of POM macromolecules, whose partial binding in the molecular structure of PLA is the reason for the formation of an irregular crystal structure characterized by a lower melting point. The phenomenon of partial miscibility is also confirmed by the cooling stage plots, where for the POM/PLA (25/75) sample the crystallization onset temperature for the POM phase can be seen to be shifted from 150 to 140 °C. A similar decrease in the crystallization temperature was observed by Ye et al. [35]. The second melting peak was detected at 178 °C and represents the PLA crystalline structure, and the peak for the pure PLA sample was detected at 170 °C. This large temperature shift was possible due to the formation of a more ordered crystalline structure of the PLA phase, which means that POM can be used as an effective nucleating agent for the PLA phase. The nucleation effect is also confirmed by the cooling thermograms where the sharp exothermic peak is observed at 105 °C.

**Figure 13:**
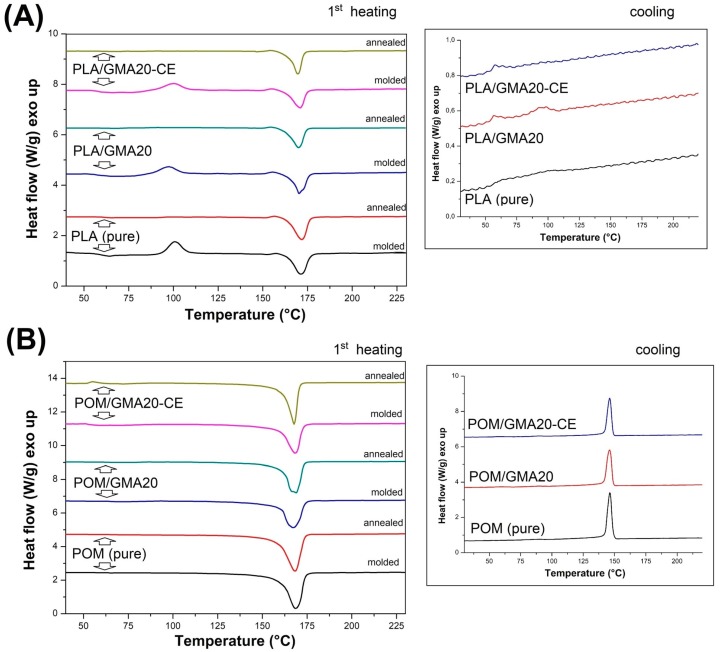
First heating and cooling for (**A**) PLA- and (**B**) POM-based blends.

The list of thermograms shown in Figure 13 includes the PLA- and POM-based samples, before and after the introduction of the impact modifier and chain extender. The first heating plots include both as-molded and annealed samples, while the cooling thermogram presents the thermal behavior of the as-molded samples. The plot for the PLA-based samples suggests clearly that annealing significantly increases the content of the crystalline phase of PLA. This is confirmed by the disappearance of the exothermic cold crystallization peak, which for all the injection molded samples occurs at around 100 °C. Table 2 presents basic thermal properties; for the pure PLA samples the results indicate an increase in the level of crystallinity to over 45% from the initial 9%, which is consequently the main reason for the significant increase in the stiffness of this type of sample. Interestingly, the proportion of the crystalline phase after the injection process varies greatly between the pure and modified PLA samples, indicating a decrease to less than 1% for the samples with the addition of EBA and CE. The main reason for this fact is the binding of a significant part of the PLA chains at the PLA–EBA interface, which is caused by the presence of active glycidyl methacrylate groups in the composition of the used impact modifier. Cooling thermograms indicate a very low level of DSC signal differences for the PLA samples, which can be seen through very slight differences on the heat flow scale, which in this situation result in a significant signal noise. The only notable signal event refers to the signal curve deflection being close to 60 °C, which relates to the glass temperature transition. There is a small crystallization peak at 100 °C which is visible for the pure PLA and PLA/GMA20 samples; however, the introduction of CE makes it disappear. Unlike with PLA, the curves for POM confirm the lack of significant structural changes after the annealing treatment. The crystallinity level for all samples stays at a similar, high level (40%) for all samples. The introduction of the EBA and CE modifier did not change any of the observed characteristics, which confirms the lack of structural changes within the POM matrix caused by the applied modifications.

**Figure 14:**
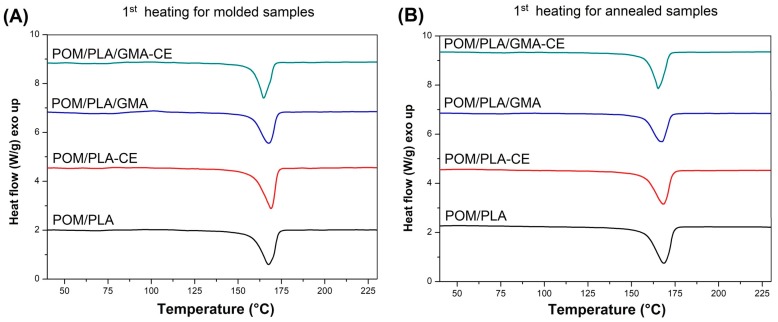

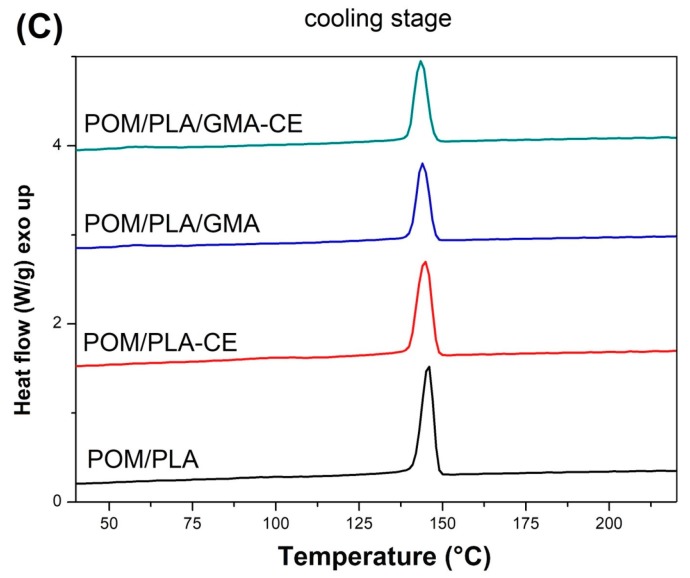
First heating thermograms for polymer blends: molded (**A**) and annealed (**B**) samples, and cooling stage (**C**).

The DSC plots collected in Figure 14 show the thermograms for the modified POM/PLA (50/50) blends. DSC signals for the prepared polymer blends indicate the dominant effect of POM thermal behavior which results from the high tendency to form the crystalline phase by this polymer. In addition to the clear cold crystallization peaks for the injected samples, the course of the rest of the graphs is very similar to pure POM, both during the heating and cooling of samples. For a more detailed analysis, selected values are summarized in Table 2, which allows for the capturing of certain differences between individual materials. Since previous DSC measurements have shown that the crystallinity of POM does not change due to changes in its composition, the calculation focused on the level of the PLA crystallinity, assuming that the crystallinity of the polyoxymethylene phase would be constant and reach 40%, with a melting enthalpy of 130 J/g. Taking all this into account, the crystallinity of PLA in the POM-mixed systems is largely dependent on the applied modification. For the unmodified samples, the content of the crystalline phase was estimated at over 22%, suggesting the possibility of active nucleation of the PLA crystalline phase by the previously formed spherulites of the POM crystalline phase. The highest level of crystallinity—over 37%—can be observed for the blends with the addition of CE, which is probably related to the partial crosslinking of the PLA structure. This phenomenon results in the formation of active nucleation centers at the points of the polymer network looping. Samples modified with the EBA–GMA elastomer do not show such an increase and the level of PLA crystallinity is slightly higher than for the original unmodified blend. This behavior may indicate the absence of the nucleating mechanism and binding of polylactide chains at the PLA–EBA interface. A subsequent decrease in crystallinity for the CE-modified samples confirms the presence of high interactions at the PLA–EBA interface. Interestingly, the results obtained for the second heating step indicate a significant decrease in the kinetics of the PLA crystallization, which can be observed through the increase in the enthalpy field of cold crystallization. This suggests the possibility of an additional process of crosslinking of the PLA structure during the DSC test, which may be related to the maximum measurement temperature of 230 °C.

**Figure 15:**
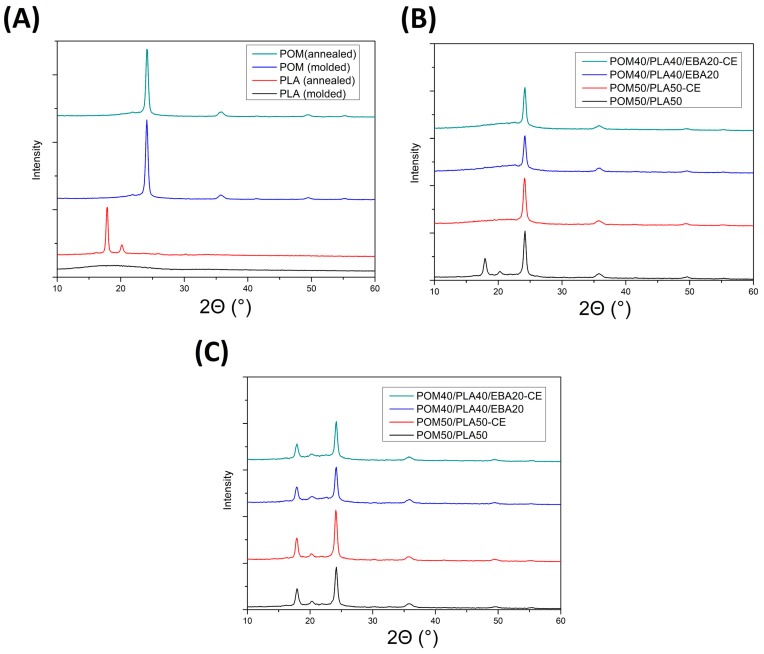
Wide-angle X-ray diffraction (WAXS) plots for (**A**) pure PLA and POM (molded and annealed), (**B**) molded POM/PLA blends, and (**C**) annealed POM/PLA blends.

WAXS plots showed in Figure 15 present diffractograms for different types of prepared samples. For the purpose of comparison Figure 15A presents the results for the pure PLA- and POM-based samples before and after the annealing procedure. A large difference in structure behavior can be seen for the PLA samples, where the amorphous structure of the as-molded sample was transformed into a highly crystalline material. The two visible diffraction peaks (110/200) and (203) at 16.8 and 19.2° 2θ, respectively, correspond with the α-type PLA crystalline phase [63,64,65].

The WAXS pattern for the POM-based samples reveals two crystal diffraction peaks at 2θ = 23° which correspond to the (100) reflection of the POM hexagonal crystals. There were no changes observed in the XRD diffractograms for the as-molded and annealed POM samples, confirming the initial high crystallinity of the POM structure and the lack of significant changes after the thermal treatment. The WAXS patterns in the next plots correspond to the modified POM/PLA blends and are shown in Figure 15B,C for the as-molded and annealed samples, respectively. As can be seen for the reference POM50/PLA50 sample, the presence of PLA (110/200) and (203) diffraction peaks confirms the improved crystallinity of the PLA phase in the presence of the POM phase. This type of behavior is not characteristic of the usually slowly crystallizing PLA phase. This is especially true for the injection-molded samples, where due to the rapid cooling of the outer surface the polymer chains stay mostly in the amorphous form. Interestingly, for the modified blends the PLA characteristic XRD crystalline phase signals are not detected and only the POM characteristic peaks can be detected. As opposed to the DSC tests, where the sample is prepared from the specimen cross-section, the WAXS measurements present the results from the sample surface. This means that for the modified POM/PLA blends, where the crystallinity calculated from the DSC measurements was found to be quite high (19–37%), the crystalline phase content in the sample cross-section can be seen to have a very large gradient. The annealing treatment (Figure 15C) once again reveals the presence of the PLA characteristic peaks, which means that the presence of GMA-functionalized modifiers does not suppress the PLA crystallization ability; however, the crystallization kinetic might be reduced, as was concluded based on the DSC results.

The results of a comparison between the DSC and WAXS measurements reveal some interesting behavior for the POM/PLA blends. The PLA-based samples were characterized by a very low crystallinity level (typical for the high cooling rates occurring during the injection molding), while for the POM-based materials the formation of a highly crystalline structure was observed. The expected test result for the POM/PLA blend was the nucleating effect of POM on the PLA crystalline phase, which was reported for the blends containing a low percentage of POM (up to 5%) [32]; however, for other types of POM-rich blends some studies have indicated the binding of the PLA chain in the amorphous form [33,35,66]. In our study, crystallinity analysis was carried out on the basis of a comparison between the DSC and WAXS measurement results, which provided a broader view of the POM/PLA system crystallization phenomenon. The DSC studies show a significant increase in the level of crystallinity for all blends, including after modification. However, as the WAXS results show, the addition of EBA and CE reactive components decreased the PLA crystallization kinetics, resulting in the occurrence of the amorphous structure of PLA on the surface of the modified samples. The kinetics of crystallization may be the key aspect determining the thermomechanical properties of POM/PLA blends, and therefore require future research.

## 4. Conclusions

The study confirms the partial miscibility of POM/PLA blend systems. Consequently, unmodified POM/PLA blends do not require any additional compatibilization. Further research into the modification of impact properties has demonstrated high efficiency in the application of the EBA/CE system. It is worth noting that in the case of reference tests conducted for pure POM and PLA, very low effectiveness of the use of used additives for POM was shown. In the case of PLA, the effectiveness of reactive blending was confirmed by most of the conducted tests. The presence of PLA in the used blend system resulted in improved compatibility at the matrix–EBA interface, which in turn resulted in a reduction in the size of EBA droplets and improved the impact strength and elongation at break values of the samples. The analysis of DMTA thermomechanical properties indicated some changes in heat resistance. However, the results of HDT and VST measurements were inconclusive because only for the VST measurements was a significant increase in thermal resistance shown, and in order to increase the sample heat deflection temperature it was observed that it is necessary to anneal the material. Analysis of DSC and WAXS results showed that in the case of the prepared POM/PLA blends, the increase in PLA crystallinity may be inhibited by the use of reactive compatibilization methods.

## Figures and Tables

**Table 1:** The results of thermomechanical measurements, namely, heat deflection temperature (HDT) and Vicat softening temperature (VST).

Sample	HDT (1.8 MPa) (°C)		VST (10 N) (°C)	
	Molded	Annealed	Molded	Annealed
Unmodified blends				
PLA	60.5 ± 1.0	74.7 ± 2.5	66.1 ± 0.2	152.4 ± 0.3
POM	90.6 ± 1.5	105.4 ± 1.3	164.1 ± 0.1	164.7 ± 0.1
POM/PLA (75/25)	60.3 ± 3.5	92.7 ± 2.6	163.2 ± 0.1	160.3 ± 0.7
POM/PLA (50/50)	53.3 ± 0.7	84.9 ± 6.2	157.9 ± 0.2	158.6 ± 0.9
POM/PLA (25/75)	54.8 ± 0.9	84.6 ± 2.4	157.6 ± 0.2	154.6 ± 0.4
EBA-modified POM and PLA resin				
POM/EBA20	73.6 ± 1.5	96.1 ± 0.5	160.9 ± 0.1	161.0 ± 0.7
POM/EBA20-CE	72.5 ± 2.0	79.2 ± 4.2	160.9 ± 0.1	161.9 ± 0.8
PLA/EBA20	58.5 ± 0.4	66.6 ± 0.3	64.1 ± 0.4	128.6 ± 0.7
PLA/EBA20-CE	59.4 ± 0.5	66.0 ± 0.4	65.2 ± 0.9	132.2 ± 0.4
Modified POM/PLA blends				
POM50/POM50-CE	53.6 ± 0.1	83.4 ± 8.3	157.9 ± 0.2	157.5 ± 0.5
POM40/PLA40/EBA20	52.2 ± 0.1	62.4 ± 1.3	148.6 ± 0.4	144.9 ± 0.6
POM40/PLA40/EBA20-CE	52.3 ± 0.6	61.8 ± 1.7	146.3 ± 0.6	147.3 ± 0.3

**Table 2:** Basic thermal properties of the prepared materials for the as-molded and annealed samples.

Sample	Molded			Annealed		
	$\Delta H_{cc}\;(J/g)$	$\Delta H_{m}\;(J/g)$	$X_{c}\;(\%)$	$\Delta H_{cc}\;(J/g)$	$\Delta H_{m}\;(J/g)$	$X_{c}\;(\%)$
PLA-based blends						
PLA	33.8	42.6	9.4	-	43.7	45.9
PLA/EBA20	32.6	33.3	0.9	-	35.2	46.4
PLA/EBA20+CE	31.6	32.1	0.7	-	33.7	44.9
POM-based blends						
POM	-	130.5	40.0	-	131.5	40.3
POM/EBA20	-	103.2	39.6	-	107.6	41.2
POM/EBA20+CE	-	96.8	37.1	-	101.8	39.0
POM/PLA-based blends (PLA phase crystallinity)						
POM50/PLA50	11.4	87.2	22.7 *	-	101.6	76.5 *
PLA50/PLA50+CE	10.2	93	37.5 *	-	91.5	55.0 *
PLA540/PLA40/EBA20	15.5	76.6	23.6 *	-	76.6	64.2 *
PLA540/PLA40/EBA20+CE	13.6	73	19.1 *	-	80.7	74.9 *

$\Delta H_{cc}$, cold crystallization enthalpy; $\Delta H_{m}$, melting enthalpy; $X_{c}$, crystallinity. * calculated PLA crystallinity, assuming constant crystallinity of the POM phase.

## References

[1] Oh H.J., Lee D.J., Lee C.G., Jo K.Y., Lee D.H., Song Y.S., Youn J.R. (2013). Warpage analysis of a micro-molded parts prepared with liquid crystalline polymer based composites. Compos. Part A Appl. Sci. Manuf..

[2] Jiang L., Wolcott M.P., Zhang J. (2006). Study of biodegradable polylactide/poly(butylene adipate-*co*-terephthalate) blends. Biomacromolecules.

[3] Wang L.-F., Rhim J.-W., Hong S.-I. (2016). Preparation of poly(lactide)/poly(butylene adipate-*co*-terephthalate) blend films using a solvent casting method and their food packaging application. LWT—Food Sci. Technol..

[4] Chiu H., Huang S., Chen Y., Kuo M., Chiang T., Chang C., Wang Y. (2013). Heat Treatment Effects on the Mechanical Properties and Morphologies of Poly(lactic acid)/Poly(butylene adipate-*co*-terephthalate) Blends. Int. J. Polym. Sci..

[5] Nofar M., Maani A., Sojoudi H., Heuzey M.C., Carreau P.J. (2015). Interfacial and rheological properties of PLA/PBAT and PLA/PBSA blends and their morphological stability under shear flow. J. Rheol..

[6] Nofar M., Tabatabaei A., Sojoudiasli H., Park C.B., Carreau P.J., Heuzey M.-C., Kamal M.R. (2017). Mechanical and bead foaming behavior of PLA-PBAT and PLA-PBSA blends with different morphologies. Eur. Polym. J..

[7] Takayama T., Todo M., Tsuji H. (2011). Effect of annealing on the mechanical properties of PLA/PCL and PLA/PCL/LTI polymer blends. J. Mech. Behav. Biomed. Mater..

[8] Odent J., Habibi Y., Raquez J.M., Dubois P. (2013). Ultra-tough polylactide-based materials synergistically designed in the presence of rubbery ε-caprolactone-based copolyester and silica nanoparticles. Compos. Sci. Technol..

[9] Di Lorenzo M.L., La Pietra P., Errico M.E., Righetti M.C., Angiuli M. (2007). Poly(butylene terephthalate)/poly(ε-caprolactone) blends: Miscibility and thermal and mechanical properties. Polym. Eng. Sci..

[10] Yew G.H., Mohd Yusof A.M., Mohd Ishak Z.A., Ishiaku U.S. (2005). Water absorption and enzymatic degradation of poly(lactic acid)/rice starch composites. Polym. Degrad. Stab..

[11] Wang H., Sun X., Seib P. (2001). Strengthening blends of poly(lactic acid) and starch with methylenediphenyl diisocyanate. J. Appl. Polym. Sci..

[12] Lv S., Gu J., Cao J., Tan H., Zhang Y. (2015). Effect of annealing on the thermal properties of poly(lactic acid)/starch blends. Int. J. Biol. Macromol..

[13] Wu N., Zhang H. (2017). Mechanical properties and phase morphology of super-tough PLA/PBAT/EMA-GMA multicomponent blends. Mater. Lett..

[14] Yuryev Y., Mohanty A.K., Misra M. (2016). A New Approach to Supertough Poly(lactic acid): A High Temperature Reactive Blending. Macromol. Mater. Eng..

[15] Zhang K., Nagarajan V., Misra M., Mohanty A.K. (2014). Supertoughened Renewable PLA Reactive Multiphase Blends System: Phase Morphology and Performance. ACS Appl. Mater. Interfaces.

[16] (2007). NatureWorks Technology Focus Report: Blends of PLA with Other Thermoplastics. https://www.natureworksllc.com/~/media/Files/NatureWorks/Technical-Documents/Properties-Documents/PropertiesDocument_Blends-of-Ingeo-with-other-thermoplastics_pdf.pdf.

[17] Wang Y.L., Hu X., Li H., Ji X., Li Z.M. (2010). Polyamide-6/poly(lactic acid) blends compatibilized by the maleic anhydride grafted polyethylene-octene elastomer. Polym. Plast. Technol. Eng..

[18] Pai F.C., Lai S.M., Chu H.H. (2013). Characterization and properties of reactive poly(lactic acid)/polyamide 610 biomass blends. J. Appl. Polym. Sci..

[19] Zolali A.M., Heshmati V., Favis B.D. (2017). Ultratough co-continuous PLA/PA11 by interfacially percolated poly(ether-*b*-amide). Macromolecules.

[20] Di Lorenzo M.L., Rubino P., Cocca M. (2014). Isothermal and non-isothermal crystallization of poly(l-lactic acid)/poly(butylene terephthalate) blends. J. Appl. Polym. Sci..

[21] Chang B.P., Mohanty A.K., Misra M. (2018). Tuning the compatibility to achieve toughened biobased poly(lactic acid)/poly(butylene terephthalate) blends. RSC Adv..

[22] Samthong C., Deetuam C., Yamaguchi M., Praserthdam P., Somwangthanaroj A. (2016). Effects of size and shape of dispersed poly(butylene terephthalate) on isothermal crystallization kinetics and morphology of poly(lactic acid) blends. Polym. Eng. Sci..

[23] Di Lorenzo M.L., Rubino P., Cocca M. (2013). Miscibility and properties of poly(l-lactic acid)/poly(butylene terephthalate) blends. Eur. Polym. J..

[24] You X., Snowdon M.R., Misra M., Mohanty A.K. (2018). Biobased Poly(ethylene terephthalate)/Poly(lactic acid) Blends Tailored with Epoxide Compatibilizers. ACS Omega.

[25] Phuong V.T., Coltelli M.B., Cinelli P., Cifelli M., Verstichel S., Lazzeri A. (2014). Compatibilization and property enhancement of poly(lactic acid)/polycarbonate blends through triacetin-mediated interchange reactions in the melt. Polymer.

[26] Lin L., Deng C., Lin G., Wang Y. (2014). Mechanical Properties, Heat Resistance and Flame Retardancy of Glass Fiber-Reinforced PLA-PC Alloys Based on Aluminum Hypophosphite. Polym. Plast. Technol. Eng..

[27] Lin L., Deng C., Lin G.-P., Wang Y.-Z. (2015). Super Toughened and High Heat-Resistant Poly(lactic acid) (PLA)-Based Blends by Enhancing Interfacial Bonding and PLA Phase Crystallization. Ind. Eng. Chem. Res..

[28] Dong W., He M., Wang H., Ren F., Zhang J., Zhao X., Li Y. (2015). PLLA/ABS blends compatibilized by reactive comb polymers: Double Tgdepression and significantly improved toughness. ACS Sustain. Chem. Eng..

[29] Vadori R., Misra M., Mohanty A.K. (2016). Sustainable biobased blends from the reactive extrusion of polylactide and acrylonitrile butadiene styrene. J. Appl. Polym. Sci..

[30] Vadori R., Misra M., Mohanty A.K. (2017). Statistical optimization of compatibilized blends of poly(lactic acid) and acrylonitrile butadiene styrene. J. Appl. Polym. Sci..

[31] Guo X. (2012). Investigation of poly(lactic acid)/polyoxy methylene blends: Crystallization behavior and heat resistance. Ph.D. Thesis.

[32] Guo X., Liu H., Zhang J., Huang J. (2014). Effects of Polyoxymethylene as a Polymeric Nucleating Agent on the Isothermal Crystallization and Visible Transmittance of Poly(lactic acid). Ind. Eng. Chem. Res..

[33] Guo X., Zhang J., Huang J. (2015). Poly(lactic acid)/polyoxymethylene blends: Morphology, crystallization, rheology, and thermal mechanical properties. Polymer.

[34] Qiu J., Xing C., Cao X., Wang H., Wang L., Zhao L., Li Y. (2013). Miscibility and double glass transition temperature depression of poly(l-lactic acid) (PLLA)/poly(oxymethylene) (POM) blends. Macromolecules.

[35] Ye L., Qiu J., Wu T., Shi X., Li Y. (2014). Banded spherulite templated three-dimensional interpenetrated nanoporous materials. RSC Adv..

[36] Mathurosemontri S., Thumsorn S., Hiroyuki H. (2016). Tensile properties modification of ductile polyoxymethylene/poly(lactic acid) blend by annealing technique. Proc. ANTEC, Indianap..

[37] Ye L., Shen J., Xie K., Li Z.-X., Li Y. (2018). Replicated Banded Spherulite: Microscopic Lamellar-assembly of Poly(L-lactic acid) Crystals in the Poly(oxymethylene) Crystal Framework. Chinese J. Polym. Sci..

[38] Barczewski M., Matykiewicz D., Andrzejewski J. (2015). Effect of heterogeneous nucleation on isotactic polypropylene-polyoxymethylene blends properties and miscibility. Macromol. Res..

[39] Lam K.L., Bakar A.A., Mohd Ishak Z.A., Karger-Kocsis J. (2004). Amorphous Copolyester/Polyoxymethylene Blends: Thermal, Mechanical and Morphological Properties. Kautsch. Gummi Kunstst..

[40] Tang W., Wang H., Tang J., Yuan H. (2013). Polyoxymethylene/thermoplastic polyurethane blends compatibilized with multifunctional chain extender. J. Appl. Polym. Sci..

[41] Jiao Q., Shen J., Ye L., Li Y., Chen H. (2019). Poly(oxymethylene)/poly(butylene succinate) blends: Miscibility, crystallization behaviors and mechanical properties. Polymer.

[42] Yuryev Y., Mohanty A.K., Misra M. (2016). Novel super-toughened bio-based blend from polycarbonate and poly(lactic acid) for durable applications. RSC Adv..

[43] Walha F., Lamnawar K., Maazouz A., Jaziri M. (2016). Rheological, Morphological and Mechanical Studies of Sustainably Sourced Polymer Blends Based on Poly(Lactic Acid) and Polyamide 11. Polymers.

[44] Wang S., Pang S., Pan L., Xu N., Li T. (2016). Isothermal cold crystallization, heat resistance, and tensile performance of polylactide/thermoplastic polyester elastomer (PLA/TPEE) blends: Effects of annealing and reactive compatibilizer. Polymers.

[45] Pan Y., Liu X., Shi S., Liu C., Dai K., Yin R., Schubert D.W., Zheng G., Shen C. (2016). Annealing Induced Mechanical Reinforcement of Injection Molded iPP Parts. Macromol. Mater. Eng..

[46] Wang J., Guo J., Li C., Yang S., Wu H., Guo S. (2014). Crystallization kinetics behavior, molecular interaction, and impact-induced morphological evolution of polypropylene/poly(ethylene-co-octene) blends: Insight into toughening mechanism. J. Polym. Res..

[47] Cocca M., Androsch R., Righetti M.C., Malinconico M., Di Lorenzo M.L. (2014). Conformationally disordered crystals and their influence on material properties: The cases of isotactic polypropylene, isotactic poly(l-butene), and poly(l-lactic acid). J. Mol. Struct..

[48] Wei Z., Song P., Zhou C., Chen G., Chang Y., Li J., Zhang W., Liang J. (2013). Insight into the annealing peak and microstructural changes of poly(l-lactic acid) by annealing at elevated temperatures. Polymer.

[49] Garlotta D. (2001). A Literature Review of Poly(Lactic Acid). J. Polym. Environ..

[50] Fischer E.W., Sterzel H.J., Wegner G. (1973). Investigation of the structure of solution grown crystals of lactide copolymers by means of chemical reactions. Kolloid-Z. Z. Polym..

[51] Kumar G., Neelakantan N.R., Subramanian N. (1995). Polyacetal and thermoplastic polyurethane elastomer toughened polyacetal: Crystallinity and fracture mechanics. J. Mater. Sci..

[52] Pielichowska K. (2012). The influence of molecular weight on the properties of polyacetal/ hydroxyapatite nanocomposites. Part 1. Microstructural analysis and phase transition studies. J. Polym. Res..

[53] Benwood C., Anstey A., Andrzejewski J., Misra M., Mohanty A.K. (2018). Improving the Impact Strength and Heat Resistance of 3D Printed Models: Structure, Property, and Processing Correlationships during Fused Deposition Modeling (FDM) of Poly(lactic acid). ACS Omega.

[54] Huang T., Yamaguchi M. (2017). Effect of cooling conditions on the mechanical properties of crystalline poly(lactic acid). J. Appl. Polym. Sci..

[55] Pan Y., Liu X., Kaschta J., Liu C., Schubert D.W. (2017). Reversal phenomena of molten immiscible polymer blends during creep-recovery in shear. J. Rheol..

[56] Zeng J.B., Li K.A., Du A.K. (2015). Compatibilization strategies in poly(lactic acid)-based blends. RSC Adv..

[57] Bouzouita A. (2016). Elaboration of Polylactide-Based Materials for Automotive Application: Study of Structure-Process-Properties Interactions Amani Bouzouita. Ph.D. Thesis.

[58] Bouzouita A., Samuel C., Notta-Cuvier D., Odent J., Lauro F., Dubois P., Raquez J.-M. (2016). Design of highly tough poly(l-lactide)-based ternary blends for automotive applications. J. Appl. Polym. Sci..

[59] Ai X., Li X., Yu Y., Pan H., Yang J., Wang D., Yang H., Zhang H., Dong L. (2019). The Mechanical, Thermal, Rheological and Morphological Properties of PLA/PBAT Blown Films by Using Bis(tert-butyl dioxy isopropyl) Benzene as Crosslinking Agent. Polym. Eng. Sci..

[60] Jiang G., Huang H.X., Chen Z.K. (2011). Rheological responses and morphology of polylactide/linear low density polyethylene blends produced by different mixing type. Polym. Plast. Technol. Eng..

[61] Li K., Peng J., Turng L.-S., Huang H.-X. (2011). Dynamic rheological behavior and morphology of polylactide/poly(butylenes adipate-co-terephthalate) blends with various composition ratios. Adv. Polym. Technol..

[62] Han C.D., Chuang H.-K. (1985). Criteria for rheological compatibility of polymer blends. J. Appl. Polym. Sci..

[63] Wu C.P., Wang C.C., Chen C.Y. (2015). Enhancing the PLA Crystallization Rate and Mechanical Properties by Melt Blending with Poly(styrene-butadiene-styrene) Copolymer. Polym. Plast. Technol. Eng..

[64] Bai H., Huang C., Xiu H., Zhang Q., Fu Q. (2014). Enhancing mechanical performance of polylactide by tailoring crystal morphology and lamellae orientation with the aid of nucleating agent. Polymer.

[65] Barrau S., Vanmansart C., Moreau M., Addad A., Stoclet G., Lefebvre J.M., Seguela R. (2011). Crystallization behavior of carbon nanotube-polylactide nanocomposites. Macromolecules.

[66] Li J., Wang Y., Wang X., Wu D. (2019). Development of Polyoxymethylene/Polylactide Blends for a Potentially Biodegradable Material: Crystallization Kinetics, Lifespan Prediction, and Enzymatic Degradation Behavior. Polymers.

